# The Italian National External Quality Assessment Program in Molecular Genetic Testing: Results of the VII Round (2010-2011)

**DOI:** 10.1155/2013/739010

**Published:** 2013-01-29

**Authors:** F. Censi, F. Tosto, G. Floridia, M. Marra, M. Salvatore, A. M. Baffico, M. Grasso, M. A. Melis, E. Pelo, P. Radice, A. Ravani, C. Rosatelli, N. Resta, S. Russo, M. Seia, L. Varesco, V. Falbo, D. Taruscio

**Affiliations:** ^1^National Centre of Rare Diseases, Istituto Superiore di Sanità, 00161 Rome, Italy; ^2^E. O. Ospedali Galliera, S.C. Laboratorio di Genetica, 16128 Genova, Italy; ^3^Dipartimento di Scienze Biomediche e Biotecnologie, Università di Cagliari, 09121 Cagliari, Italy; ^4^SOD Diagnostica, AOU Careggi, 50134 Firenze, Italy; ^5^Unit of Molecular Bases of Genetic Risk and Genetic Testing, Department of Preventive and Predictive Medicine, Fondazione IRCCS Istituto Nazionale dei Tumori, 20133 Milan, Italy; ^6^Department of Reproduction and Growth, Operative Unit of Medical Genetics, University Hospital S. Anna, Ferrara, Italy; ^7^Department of Public Health, Clinical and Molecular Medicine, University of Cagliari, 09121 Cagliari, Italy; ^8^Dipartimento di Scienze Biomediche ed Oncologia Umana, University of Bari, 70124 Bari, Italy; ^9^Laboratory of Molecular Genetics, Istituto Auxologico Italiano, Cusano Milanino, 20135 Milano, Italy; ^10^Laboratorio di Genetica Medica, Fondazione IRCCS Policlinico Ca' Granda Ospedale, Milano, Italy; ^11^Unit of Hereditary Cancer, Department of Epidemiology, Prevention and Special Functions, Istituto Nazionale per la Ricerca sul Cancro (IST), Genova, Italy

## Abstract

Since 2001 the Istituto Superiore di Sanità established a quality assurance programme for molecular genetic testing that covers four pathologies: Cystic Fibrosis (CF), Beta Thalassemia (BT), Fragile X Syndrome (FX), and Familial Adenomatous Polyposis Coli (APC). Since 2009 this activity is an institutional activity and participation is open to both public and private laboratories. Seven rounds have been performed until now and the eighth is in progress. Laboratories receive 4 DNA samples with mock clinical indications. They analyze the samples using their routine procedures. A panel of assessors review the raw data and the reports; all data are managed through a web utility. In 2010 the number of participants was 43, 17, 15, 5 for CF, BT, FX, APC schemes respectively. Genotyping results were correct in 96%, 98.5%, 100%, and 100% of CF, BT, FX, and APC samples, respectively. Interpretation was correct in 74%, 91%, 88%, and 60% of CF, BT, FX, and APC reports, respectively; however in most of them it was not complete but a referral to genetic counseling was given. Reports were satisfactory in more than 60% of samples in all schemes. This work presents the 2010 results in detail comparing our data with those from other European schemes.

## 1. Introduction 

Since the human genome sequencing was completed, the number of diseases for which genetic tests are available has grown rapidly (2500 diseases for which genetic tests were available in 2011—http://www.ncbi.nlm.nih.gov/projects/GeneTests/static/whatsnew/labdirgrowth.shtml).

Genetic tests are unique in their kind; they are performed only once in the life of a patient because their outcome never changes. Therefore an error may have harmful consequences on the choice of clinical/therapeutic planning and can significantly affect the life choices of the patients and their family.

Laboratories that perform genetic tests are required to work to very high quality standards; monitoring such laboratories is an obligation for the National Health System as part of its mandate to protect the health and quality of life of citizens.

The role of External Quality Assessments (EQA) in ensuring good laboratory practice is recognized at national and international level [1–5]. EQA schemes are the main tools for measuring the quality of laboratory results, for maintaining confidence in molecular genetic tests, and for implementing the standards of quality assurance [6–9]. A number of initiatives were taken internationally to improve quality in genetic testing services, for example, Cystic Fibrosis Quality Network and EMQN in Europe, and CAP in the USA [10, 11]. 

In 2001 the Italian National Centre for Rare Diseases of the Istituto Superiore di Sanità (ISS, Rome) established EQA schemes for both molecular genetic testing and classical cytogenetic. In particular, for molecular genetic testing, it offers specific EQA schemes for 4 diseases: Cystic Fibrosis (*CFTR* gene) (CF), Beta Thalassemia (*HBB* gene) (BT), Fragile X-Syndrome (*FMR1* gene) (FX), and Familial Adenomatous Polyposis Coli (*APC* gene) (APC) [12]. 

The Italian EQA (IEQA) has primarily an educational role and it aims to improve the quality of genetic tests used in clinical practice [12].

Until now seven rounds have been completed and overall 91 different laboratories have been monitored in the context of the (IEQA). National experts have assessed laboratory performance on genotyping, interpretation, and reporting of test results for a total number of 3158 samples.

A web utility was developed in 2008 to support this activity; it represents a computer interface, among the ISS, the laboratories, and the assessors, that facilitates communication, simplifies data archiving, and minimizes paper usage.

In 2009 (VII round, 2010) the activity was published in the Official Bulletin of the Italian Republic [13]. Participation to IEQA is voluntary and open to both public and private laboratories; laboratories pay a fee to participate.

During the first six rounds the assessment focused in particular on genotyping results and on completeness of reports to evaluate technical ability and harmonize reports among Italian laboratories. Results show that genotyping is in general of good quality whereas reporting presents much larger variations between laboratories and generally a lack of information [14–17]. In the seventh round assessors focused their attention on the ability of laboratories to accurately detect mutations and, in particular, on the ability to interpret the results. 

In this work we describe the results of the seventh round of IEQA.

## 2. Materials and Methods

### 2.1. Organization of the IEQA

IEQA is organized and coordinated by the National Centre of Rare Diseases of the ISS [12]. Participation is open to both public and private laboratories. Since 2008 the activity is supported by the web utility. 

Schemes are strictly anonymous and the identity of laboratories is known only to the ISS. The IEQA scheme organizer and national experts provide advice on the scientific context of the scheme and take decisions and educational actions for the development of the program.

In every scheme ISS provides 4 validated samples of genomic DNA for each round; all samples are distributed with mock data identifications, mock clinical information, and technical data. 

Laboratories are asked to test samples using their routine protocols and to provide results of genotyping (raw data) and a full interpretative report in their normal laboratory style by a given deadline: 60 days for CF, BT, and FX; 90 days for APC.

Laboratory results are evaluated by assessors according to established criteria and results are available to laboratories in the reserved area on the web utility.

### 2.2. Sample Collection and Validation

The genomic DNA samples were obtained from peripheral blood and lymphoblastoid cell lines collected, respectively, by a clinical hospital (Presidio Ospedaliero Microcitemico, Cagliari) (only for the BT scheme) and by biobanks (Galliera Genetic Bank, Genova, and Coriell Cell Repository, Camden, NJ). Two independent working units in the ISS were responsible for DNA samples processing and validation [12].

Mutations carried by samples are validated in the ISS by routine methods: (1) direct sequencing for APC [17]; (2) PCR and Southern Blot for FX [15]; (3) PCR, sequencing and Reverse Dot Blot (RDB) for BT [16]; (4) PCR and RDB Kit according to manufacturing protocols (INNO-LiPA *CFTR*19 and INNO-LiPA *CFTR*17+Tn, Innogenetics, Belgium) for CF. Each laboratory received four aliquots of 7 *μ*g, 7 *μ*g, 20 *μ*g, and 40 *μ*g of validated DNA for the CF, BT, APC, and FX scheme, respectively.  Table 1 lists samples with mock clinical data and mutations submitted during the VII round.

### 2.3. Web Utility

The web utility was developed in 2008 and was designed to simplify communications and data sharing among ISS, laboratories, and assessors. 

Laboratories receive an identification code (ID) and password (PW) to access the personal area (http://www.iss.it/site/cnmr/privato/cqtg/entry.asp) and the scheme area where they find samples data and instructions to participate in the EQA.

Upon completing the analysis of the samples, the laboratories upload the raw data (jpg format) and the reports (pdf format). 

At deadline the data are made available to the assessors who access their reserved area; they assess the results of the laboratories and write their observations in a schedule that is forwarded to the ISS via the web utility.

Final results are uploaded in the reserved area of each laboratory which, however, is informed by e-mail. A report with a summary of all anonymous results is also included in the reserved area and published on the website (http://www.iss.it/cnmr/tege/qual/cont.php?id=90&lang=1&tipo=4).

### 2.4. Assessment

National experts evaluated the laboratory results twice: first online and then in a meeting at the ISS. Assessment took into account technical performance (raw data), genotyping, interpretation, and reports. All data were treated anonymously and the identity of each laboratory is unknown to the assessors.

Following the assessment, participating laboratories received a feedback with an evaluation of the results. Since 2009 a marking system has been introduced and laboratories receive a mark for genotyping, interpretation, and reporting of results for each sample including comments or suggestions, if necessary. Until now poor performance has neither been assigned nor penalized.

### 2.5. Evaluation Criteria

Evaluation criteria have been established by the ISS and a panel of national experts taking into account the EMQN criteria, national and international best practice guidelines and publications [18–20]. 

For each scheme, three assessment topics are identified; a maximum score of 5 points is assigned to each topic for genotyping performance, and of 4 points for interpretation and for reporting. In accordance with the evaluation criteria, points are subtracted for errors and for lack of important information. The presence of raw data and reports and the correct identification of the genotype are necessary preconditions for making the assessment. If the genotyping result is not correct, assessors do not mark the interpretation and the report, but write a comment as feedback to help the laboratories to improve their performance. 

The main common elements of the evaluation criteria for all schemes are listed in Table 2; specific topics were discussed by the assessors from time to time within the framework of each scheme.

## 3. Results

Fifty-six different laboratories participated in the VII round of the IEQA: 41 laboratories affiliated with the public health system and 15 private laboratories. In particular, 43 laboratories participated for CF, 17 for BT, 15 for FX, and 5 for APC. 22 laboratories participated in more than one scheme. During this round 320 samples were analyzed and results were examined by the assessors to evaluate their performance.

### 3.1. Genotyping Results

#### 3.1.1. Cystic Fibrosis

Overall, 172 samples were sent to the laboratories by the ISS. Genotypes were correctly detected in 165/172 (96%) samples. Genotyping errors occurred in 4% of samples (Table 3). Four laboratories gave no information about the methods used to analyze 14 samples.

#### 3.1.2. Beta Thalassemia

Overall, 68 samples were sent to the laboratories by the ISS. Genotypes were correctly detected in 67/68 (98.5%) of samples. Genotyping errors occurred in 1/68 (1.5%) samples (Table 3).

#### 3.1.3. Fragile X Syndrome

Overall, 60 samples were sent to the laboratories by the ISS. Genotypes were correctly detected in 60/60 samples (100%), but errors occurred in (CGG)_*n*_ repeat quantification (Table 3). Moreover in one sample the methylation test was not reported.

#### 3.1.4. Familial Adenomatous Polyposis of the Colon (Gene *APC*)

Overall, 20 samples were sent to the laboratories by the ISS. All samples were correctly genotyped for pathogenic mutations. However one laboratory did not identify an additional gene variant present in one sample (Table 3). 

Raw data of 3 samples analyzed by the same laboratory did not have good quality.

### 3.2. Interpretation Results

**Figure 1:**
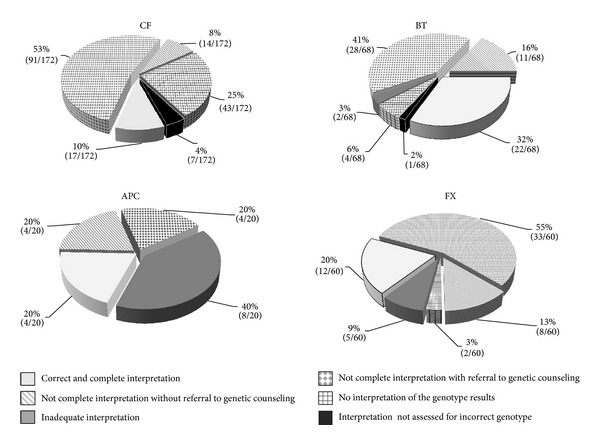
Interpretation of genotyping results.

Figure 1 shows detailed results on the assessment of genotype interpretation and includes also samples that were not assessed for incorrect genotype; Table 4 shows the information that is most commonly missing for all schemes.

#### 3.2.1. Cystic Fibrosis, Beta Thalassemia, and Fragile X Syndrome

Correct interpretation was reported in 71%, 91%, and 88% of CF, BT, and FX cases, respectively, even though a lack of information was found in the majority of them (i.e., 86%, 64%, and 77% of CF, BT, and XF reports, resp.). It has to be underlined that a referral to genetic counseling was present in most reports not complete (i.e., 87%, 72%, and 80% of CF, BT, and XF reports, resp.).

There was not any interpretation of genotyping results in 25%, 6%, and 3% of CF, BT, and FX reports, respectively; however most CF and FX reports refer to genetic counseling (i.e., 27/43 CF and 2/2 FX reports).

#### 3.2.2. Familial Adenomatous Polyposis of the Colon (Gene *APC*)

Interpretation was correct in 12/20 (60%) of reports, even though it was not complete in the majority of them, that is, 8/12 (67%). It has to be underlined that, when the interpretation was lacking information, a referral to genetic counseling was indicated in most reports, that is, 4/8 (50%).

### 3.3. Report Results

Table 5 shows information most commonly missing in the reports for all schemes.

#### 3.3.1. Cystic Fibrosis

70% of reports assessed were correct and complete; 50 reports were not evaluated for lack of interpretation or for genotyping error. Few laboratories presented incomplete reports for lack of information such as clinical indication, and ethnic or geographic origin of the patient. Only one laboratory (4 samples) sent inadequate reports for lack or unclear reporting of important information. 

#### 3.3.2. Beta Thalassemia

65% of reports assessed were correct and complete; 1 report was not assessed for incorrect genotype. Few laboratories presented incomplete reports for lack of information such as clinical indication and title of the report; only one laboratory sent inadequate reports on all their samples for lack or unclear reporting of important information.

#### 3.3.3. Fragile X Syndrome

Only 60% of reports were found to be correct and complete. Few laboratories presented incomplete reports for lack of information such as voice of clinical indication or indication of gender.

#### 3.3.4. Familial Adenomatous Polyposis of the Colon (Gene *APC*)

60% of reports were found to be correct and complete. One laboratory (4 samples) did not report clinical indications; one laboratory (4 samples) did not indicate the title of the report.

## 4. Discussion

The EQA program focuses in particular on standardizing laboratory procedures. Participation in EQA schemes provides a measure of technical, analytical, and interpretative performance. It has an educational role for laboratories and gives the opportunity to review their internal standards and policies, and also to provide advice on the updating of best practice guidelines [1, 4, 21]. Moreover participation in EQA program is essential for laboratory accreditation with the international ISO 15189 standard [7, 21]. To date, EQA programs have been also used by laboratories as a tool for improving sample processing quality, hence the assessment takes into account not only genotyping and reporting, but it also looks at other aspects such as the interpretation of results [1, 21].

The Italian molecular EQA program was established in 2001; the VII round (2010) was reviewed versus previous rounds. 

Fifty-six laboratories participated in the VII round and 320 samples were analyzed.

Genotyping was found to be quite satisfactory in general; only 4/56 laboratories in all schemes failed to correctly genotype samples: 3 for CF (4% of cases) and 1 for BT (1,5% of cases). In comparison, the error percentage for the CF scheme was higher in this round than the median error rate registered in previous rounds (0.2%) [14]. In the framework of the BT scheme, only the analysis of 1/68 samples was wrong (1.5%) and the error percentage was higher than the median error rate reported in previous rounds (0.33%) [16]. The analysis of these errors shows that techniques were generally well performed and raw data had good quality. In spite of previous rounds, where errors were mainly due to technical errors, during the VII round all errors were caused by suboptimal management of samples [14, 16].

No genotyping errors were performed by laboratories participating in the APC and FX schemes; this is a good result which reflects the outcome of the other rounds for the APC scheme and it represents an improvement in quality for the FX scheme [15, 17]. However, in the FX scheme, some genotypes were not completely defined because the number of triplets were not accurately reported in 28% of laboratory reports, and a variant, in one sample in APC scheme, was not detected by one laboratory.

Although laboratories returned acceptable analytical results in 97.5% of samples in all schemes, in the majority of reports, with variants within each schemes, the interpretation was correct but not complete. This data can be explained by the fact that during routine analysis, laboratories write out a technical report and leave it up to the genetic consultant to provide an accurate interpretation of genotype results in a separate report. In fact about 82% of laboratories who were penalized for lack of interpretation, or lack of necessary information, suggest and/or offer genetic counseling.

A comparison between our results and other published data shows a similar scenario during the first year of the interpretation survey [5, 22]. 

Like genotyping results, also report results were satisfactory for completeness of the information; during the VII round, in fact, 66% of reports on samples were good. 

The focus on IEQA for the VII round shows satisfactory quality in genotyping and reports in comparison with previous published data, but we still observe room for improvement. A closer examination of the entire process of data reporting has highlighted the need to focus attention on the interpretation of results; this aspect was neglected in previous rounds because more attention was attached to the technical approach to sample analysis in order to ensure sound genotyping results. 

All data collected within the framework of the IEQA and the European EQAs highlighted the need and the importance to carry on this activity in order to ensure adequate quality standards for the genetic tests performed in all laboratories. 

At the present time the VIII round of the IEQA is under way; there has been an increase in the number of participating laboratories, and we expect some improvements in the laboratories that participated in the VII round. 

Moreover in this round we are introducing a “poor performance” score for laboratories that make critical errors in genotype and/or interpretation and/or reports, that may significantly affect patient management. In this context our role is to contribute to improving laboratory quality through specific educational actions, improving the dialogue with laboratories and, if necessary, involving assessors and national experts in the process.

## Figures and Tables

**Table 1:** List of proposed mutations and mock clinical information.

Scheme	Identification data	Gender	Clinical information	Proposed mutations
	Irene Pettorbi 12/01/1989	F	Female of Pakistani origin affected by Cystic Fibrosis; she manifests moderate respiratory symptomatology, pancreatic sufficiency, and normal values of sweat chloride	c.3717+12191C>T/c.3717+12191C>T (3849+10KbC>T/3849+10KbC>T)
CF	Manuela Statenti 13/01/1998	F	Female with positive sweat test, mild breathing symptoms. She asks for molecular characterization for Cystic Fibrosis.	c.579+1G>T/c.489+1G>T (711+1G>T/621+1G>T)
	Sara Ulmilefa 12/04/1966	F	Female clinically healthy with child affected by Cystic Fibrosis	c.3846G>A heterozygous (W1282X heterozygous)
	Anna Ellicine 05/05/1995	F	Female with positive sweat test; her brother is affected by Cystic Fibrosis. She asks for molecular characterization	c.1521_1523delCTT/c.1657C>T (F508del/R553X)

BT	Mario Tappenti 05/09/1982	M	Affected by Beta Thalassemia major	c.20delA/c.118C>T (Bcd6(-A)/Bcd39C>T)
	Antonio Aberuste 22/01/1988	M	Affected by Beta Thalassemia major	c.118C>T/c.118C>T (Bcd39C>T/Bcd39C>T)
	Giovanni Pormitou 17/07/1987	M	Affected by Beta Thalassemia intermediate	c.20delA/c.93-21G>A (Bcd6(-A)/IVS1-110G>A)
	Elio Smantico 01/06/1976	M	Carrier of Beta Thalassemia	c.93-21G>A heterozygous (IVS1-110G>A heterozygous)

FX	Dompinti Anna 20/02/1983	F	Female, with normal phenotype, has two children and a brother affected by Fragile X Syndrome	23/200 repeats (Premutation)
	Ornicapo Irene 12/03/1983	F	Female, with normal phenotype, has a nephew and an uncle affected by Fragile X Syndrome	29/90 repeats (Premutation)
	Quezzamo Nicola 13/03/1958	M	Male, with normal phenotype, has brother and nephew with Fragile X Syndrome; suspect carrier	100 repeats (Premutation)
	Ubbronti Mario 30/12/1970	M	Male with suspect on Fragile X Syndrome	30 repeats (Wild)

APC	Anuttifo Ennio 05/02/1969	M	No clinical indication	c.4012C>T heterozygous* c.4597A>C heterozygous^#^
	Piclilma Gianni 14/02/1957	M	No clinical indication	c.1629_1630delT heterozygous
	Ordectio Mario 17/03/1995	M	No clinical indication	c.1621C>T heterozygous
	Simpieti Aldo 11/03/1977	M	No clinical indication	c.3149delC heterozygous* c.7417C>T heterozygous^#^

CF: Cystic Fibrosis; BT: Beta Thalassemia; FX: Fragile X Syndrome; APC: Familial Polyposis Adenomatous Coli. *Pathogenic mutation; ^#^additional gene variant.

**Table 2:** General evaluation criteria: items common to all schemes taken into account for assessment.

Genotyping	
Quality of raw data	
Lack of data legend	
Lack of DNA variants detection	
Correctness of the nomenclature	
Completeness of technical information	
Correctness/lack of detection rate	
Problems with counting of triplets (X-Fra)	
Lack of indication for advanced investigations when appropriate	
Interpretation	
Lack and/or inaccuracy of important information on the pathogenic role of mutation and/or reproductive risk (not for APC), and/or other important information.	
Lack of information about test validity	
Lack of genetic counseling indication if necessary	
Reporting	
General inadequacy of the report	
Inadequate language	
Lack of laboratory heading	
Lack of identification of the patient	
Clerical error in identification of the patient	
Lack of gender indication	
Lack of geographical origin of the patient where necessary	
Lack of identification number of sample	
Lack of report title	
Lack of reason for testing	
Lack of sample source	
Lack of primary sample type	
Lack of signature of the person releasing the report	
Lack of date primary sample collection and release of the report	
Lack of indication of certification/accreditation of laboratory	
Lack of page numbering	

**Table 3:** Errors performed by laboratories in genotyping detection and reporting.

CEQ scheme	*N* of laboratories performing errors	*N* of samples	Type of error	Error percentage/scheme
CF	2/43	6/172	Genotyping error: samples swap	4%
	1/43	1/172	Genotyping error: mutation correctly detected but not correctly reported (c.3484C>T instead of c.3846G>A)	
BT	1/17	1/68	Genotyping error: mutation correctly detected but not correctly reported (c.118C>T was reported in heterozygous instead of homozygous status)	1,5%
FX	5/15	17/60	Information about genotype not adequate: number of triplets absent or not clearly reported	28%
APC	1/5	1/20	Information about genotype not adequate: gene variant not reported	5%

CF: Cystic Fibrosis; BT: Beta Thalassemia; FX: Fragile X Syndrome; APC: Familial Polyposis Adenomatous Coli.

**Table 4:** Information most commonly missing in interpretation of results.

Incomplete interpretation				
Not mentioned information	CF (105)	BT (39)	FX (41)	APC (8)
Analytical sensitivity and specificity of procedures	88%	89%	20%	100%
Detection rate absent or incorrect	60%	69%	93%	0%
Indication for genetic counseling	13%	28%	20%	50%
Reproductive risk or request to test the partner	52%	28%	0%	—
Request to test parents to confirm homozygous nature of mutation	0%	18%	—	—

CF: Cystic Fibrosis; BT: Beta Thalassemia; FX: Fragile X Syndrome; APC: Familial Polyposis Adenomatous Coli. The numbers of samples that were reported with incomplete interpretations are indicated in brackets.

**Table 5:** Details of lack of information/inadequacy in reporting results.

Reporting	FC (122)	BT (67)	XF (60)	APC (20)
General inadequacy of the report	3,30%	6%	6,60%	
Inadequate language		**13,40%**	6,60%	
Lack of laboratory heading				
Lack of identification of the patient	3,30%		6,60%	
Clerical error in identification of the patient		4,40%		
Lack of gender indication			**40%**	
Lack of geographical origin of the patient where necessary	**53,20%**			
Lack of identification number of sample		4,40%		
Lack of report title	11,40%	10,40%		20%
Lack of reason for testing	**14,70%**	**10,40%**	**22%**	**20%**
Lack of sample source	1,60%		6,60%	40%
Lack of primary sample type		6%	6,60%	
Lack of signature of the person releasing the report				
Lack of date primary sample collection and release of the report			6,60%	
Lack of indication of certification/accreditation of laboratory	36%		6%	40%
Lack of page numbering	36%			

CF: Cystic Fibrosis; BT: Beta Thalassemia; FX: Fragile X Syndrome; APC: Familial Polyposis Adenomatous Coli. The numbers of evaluated reports for pathologies are indicated in brackets.

## References

[1] Libeer JC (2001). Role of external quality assurance schemes in assessing and improving quality in medical laboratories. *Clinica Chimica Acta*.

[3] OECD (2007). *Guidelines for Quality Assurance in Molecular Genetic Testing*.

[4] Hastings RJ, Howell RT (2010). The importance and value of EQA for diagnostic genetic laboratories. *Journal of Community Genetics*.

[5] Berwouts S, Girodon E, Schwarz M (2012). Improvement of interpretation in cystic fibrosis clinical laboratory reports: longitudinal analysis of external quality assessment data. *European Journal of Human Genetics*.

[6] http://www.cnmr.iss.it/lgui.

[7] Ramsden SC, Deans Z, Robinson DO (2006). Monitoring standards for molecular genetic testing in the United Kingdom, the Netherlands, and Ireland. *Genetic Testing*.

[8] Sciacovelli L, Secchiero S, Zardo L, Zaninotto M, Plebani M (2006). External quality assessment: an effective tool for clinical governance in laboratory medicine. *Clinical Chemistry and Laboratory Medicine*.

[9] Chen B, Gagnon M, Shahangian S (2009). Good laboratory practices for molecular genetic testing for heritable diseases and conditions. *MMWR Recommendations and Reports*.

[10] Richards CS, Grody WW (2003). Alternative approaches to proficiency testing in molecular genetics. *Clinical Chemistry*.

[11] Weck KE, Zehnbauer B, Datto M (2012). Molecular genetic testing for fragile X syndrome: laboratory performance on the College of American Pathologists proficiency survey (2001–2009). *Gentics in Medicine*.

[12] Taruscio D, Falbo V, Floridia G (2004). Quality assessment in cytogenetic and molecular genetic testing: the experience of the Italian Project on Standardisation and Quality Assurance. *Clinical Chemistry and Laboratory Medicine*.

[14] Salvatore M, Falbo V, Floridia G (2007). The Italian External Quality Control Programme for cystic fibrosis molecular diagnosis: 4 years of activity. *Clinical Chemistry and Laboratory Medicine*.

[15] Falbo V, Floridia G, Tosto F (2008). The Italian External Quality Assessment scheme for fragile X syndrome: the results of a 5-year survey. *Genetic Testing*.

[16] Tosto F, Salvatore M, Falbo V (2009). The Italian scheme of External Quality Assessment for beta-thalassemia: genotyping and reporting results and testing strategies in a 5-year survey. *Genetic testing and molecular biomarkers*.

[17] Censi F, Falbo V, Floridia G (2010). The Italian external quality control program for familial adenomatous polyposis of the colon: five years of experience. *Genetic Testing and Molecular Biomarkers*.

[18] Castellani C, Cuppens H, Macek M (2008). Consensus on the use and interpretation of cystic fibrosis mutation analysis in clinical practice. *Journal of Cystic Fibrosis*.

[19] Dequeker E, Stuhrmann M, Morris MA (2009). Best practice guidelines for molecular genetic diagnosis of cystic fibrosis and CFTR-related disorders - Updated European recommendations. *European Journal of Human Genetics*.

[20] Grasso M, Melis MA, Murgia A http://www.sigu.net/index.php?option=com_docman&task=cat_view&gid=46&limitstart=15.

[21] Berwouts S, Fanning K, Morris MA (2012). Quality assurance practices in Europe: a survey of molecular genetic testing laboratories. *European Journal of Human Genetics*.

[22] Touitou I, Rittore C, Philibert L, Yagüe J, Shinar Y, Aksentijevich I (2009). An international external quality assessment for molecular diagnosis of hereditary recurrent fevers: a 3-year scheme demonstrates the need for improvement. *European Journal of Human Genetics*.

